# Foodborne Trematodiasis and *Opisthorchis felineus* Acquired in Italy

**DOI:** 10.3201/eid2001.130476

**Published:** 2014-01

**Authors:** Herman F. Wunderink, Wouter Rozemeijer, Peter C. Wever, Jaco J. Verweij, Lisette van Lieshout

**Affiliations:** Leiden University Medical Center, Leiden, the Netherlands (H.F. Wunderink, J.J. Verweij, L. van Lieshout);; Tergooihospitals, Hilversum, the Netherlands (W. Rozemeijer);; Jeroen Bosch Hospital, ’s-Hertogenbosch, the Netherlands (P.C. Wever)

**Keywords:** foodborne, trematodiasis, parasites, trematodes, Opisthorchis felineus, Italy, raw fish, foodborne diseases, opisthorchiasis, fluke worms, liver fluke infections, undercooked aquatic products

**To the editor:** Opisthorchiasis comprises diverse clinical manifestations caused by infections with *Opisthorchis felineus* or *O. viverrini* liver flukes, which are transmitted by eating infected raw or undercooked fish and other aquatic products. In regions outside Western Europe where human opisthorchiasis is endemic, the disease is mainly described as being chronic and asymptomatic. Recent studies indicate cases of *O. felineus* infection in the Mediterranean region, particularly Italy (*1**–**4*). Patients with acute infection have signs/symptoms ranging from fever to hepatitis-like signs/symptoms (e.g., pain in upper right abdominal quadrant, weakness, fatigue, loss of appetite, diarrhea, weight loss); sign/symptom onset occurs ≈2–3 weeks after infection, depending on the number of ingested flukes (*2*–*4*).

Acute opisthorchiasis is a feature of *O. felineus* infection that is not often reported for other trematode infections. Opisthorchiasis is characterized by hepatosplenomegaly, abdominal tenderness, eosinophilia, chills, and fever (*2*); left untreated, it can lead to obstructive jaundice, cholangitis, cholecystitis, and intra-abdominal masses (*1*,*2*,*4*).

Transmission of *O. viverrini* mainly occurs in Southeast Asia, but *O. felineus* transmission expands further westward to parts of Western and Central Eurasia (*1*,*2*,*4*). Recent outbreaks of *O. felineus* infection have been described in Italy (*5*–*7*). In 2010, two travelers from the Netherlands who ate raw tench near Lake Bolsena in Tuscany, Italy, were infected (*8*). We describe 3 additional cases of *O. felineus* infection in Dutch travelers who ate raw fish near Lake Bolsena.

In August 2011, a 54-year-old woman in the Netherlands with no relevant medical history sought medical care for fever, chills, and myalgia lasting 2 weeks. Symptoms began after the patient returned from a vacation in Tuscany. Physical examination showed no abnormalities; her temperature was 37.4°C. Laboratory examinations showed eosinophilic leukocytosis, an elevated C-reactive protein level, and elevated liver enzyme levels (Table). 

**Table:** Clinical parameters and results of opisthorchiasis diagnostic tests for 3 Dutch travelers after visiting Tuscany, Italy, in 2011*

Patient	Symptom severity	Eosinophilia	Liver enzyme level	Microscopy finding	*Fasciola* spp. †		*Opisthorchis felineus* real-time PCR
					IFA IgG	ELISA IgG	
Index patient	Severe	Present	Elevated	*Opisthorchis* egg	1:128	<1:40	Positive, C_t_ 24.7
Travel companion 1	Moderate	Present	Elevated	*Opisthorchis* egg	1:64	<1:40	Positive, C_t_ 25.1
Travel companion 2	Moderate	Present	Elevated	Negative	<1:32	1:160	Negative

*IFA, immunofluorescence assay; C_t_, cycle threshold. †Cut-off values for *Fasciola* spp. serology: IFA IgG 1:40, ELISA IgG 1:32.

Opisthorchiasis was suspected because of the patient’s travel history and report of eating carpaccio (Italian dish made with raw fish/meat) near Lake Bolsena (*8*). A fecal sample examined by microscopy was negative for eggs, cysts, and helminths. A serum sample was tested at Leiden University Medical Center by using an in-house immunofluorescence assay and ELISA with *Fasciola* spp. antigens, which are likely to show cross-reactivity with other liver flukes (*9*). The immunofluorescence assay result was positive, but the ELISA result was negative.

To confirm the diagnosis of opisthorchiasis, we obtained another fecal sample 1 month later, and low numbers of *Opisthorchis* eggs were seen by microscopy. The sample was sent to Leiden University Medical Center, where in-house real-time PCR was performed using primers (Of350F 5′-CTC CGT TGT TGG TCC TTT GTC-3′ and Of418R 5′-AAA CAG ATT TGC ATC GAA TGC A-3′) and a detection probe (Opis372 FAM-5′-TGC CAA CAC TGG AGC CTC AAC CAA-3′-BHQ1) designed from the *O. felineus* internal transcribed spacer 2 sequence (GenBank accession no. DQ513407). This PCR amplifies and detects a 69-bp fragment within the *O. felineus* internal transcribed spacer 2 sequence. Simultaneous isolation, amplification, and detection of a standard amount of phocid herpesvirus were used for internal control of inhibition (*10*). The *O. felineus* real-time PCR result was positive (cycle threshold 24.7).

The patient had 2 travel companions with similar, but less severe, symptoms. Both had serology test results positive for *Fasciola* spp. For 1 traveler, microscopic examination revealed an *Opisthorchis* egg in a fecal specimen, and *O. felineus* real-time PCR was positive (threshold 25.1). All 3 patients were treated with praziquantel (25 mg/kg orally 3 times/d for 2 d) and completely recovered.

Foodborne trematodiasis is re-emerging and occurring in developed regions (*1*–*3*). A total of ≈8.4 million persons worldwide have opisthorchiasis, of whom ≈325,000 are in Europe (*1*). Earlier reports of human infections around Lake Bolsena did not result in complete transmission control in the region, as illustrated by the current cases.

The reference standard for diagnosing opisthorchiasis is observation of eggs in feces by microscopy. However, the sensitivity of microscopy is low, particularly in the early disease stage because egg production starts 1–3 months after exposure (*4*,*9*), and the similarity of eggs of different trematodes hampers species-specific differentiation (*1*). In addition, sensitivity of microscopy is highly observer-dependent and varies with the microscopist’s level of experience. Because most opisthorchiasis cases in Europe have low numbers of worms, at least 3 separate fecal samples should be obtained and thoroughly examined to rule out a positive diagnosis (*4*,*9*). If test results are negative, a fecal examination should be repeated after several weeks.

Specific *Opisthorchis* spp. serology tests are not available within the Netherlands, but because of known serologic cross-reactivity, antibody detection for *Fasciola* spp. can be performed if opisthorchiasis is suspected (*9*). For confirmation, an *O. felineus*–specific real-time PCR can be performed.

Although opisthorchiasis is not frequently reported in Europe, it should be considered in cases of unexplained acute febrile eosinophilic syndrome with cholestasis, especially when patients confirm the ingestion of raw or undercooked aquatic products. Furthermore, opisthorchiasis should be considered even without a relevant travel history to regions outside Europe where the disease is endemic.
