# Correction: Vol. 20, No. 1

**DOI:** 10.3201/eid2012.C12012

**Published:** 2014-12

**Keywords:** errata, erratum, correction

In the article Foodborne Trematodiasis and *Opisthorchis felineus* Acquired in Italy (H. F. Wunderink et al.), author Wouter Rozemeijer’s name was spelled incorrectly. The article has been corrected online (http://wwwnc.cdc.gov/eid/article/20/1/13-0476_article).

